# Ischemic stroke, hemorrhage, and mortality in patients with non-valvular atrial fibrillation and renal dysfunction treated with rivaroxaban: sub-analysis of the EXPAND study

**DOI:** 10.1007/s00380-021-01810-5

**Published:** 2021-03-16

**Authors:** Hirotsugu Atarashi, Shinichiro Uchiyama, Hiroshi Inoue, Takanari Kitazono, Takeshi Yamashita, Wataru Shimizu, Takanori Ikeda, Masahiro Kamouchi, Koichi Kaikita, Koji Fukuda, Hideki Origasa, Hiroaki Shimokawa

**Affiliations:** 1 Minamihachioji Hospital, Koyasu-cho 3-18-12, Hachioji, Tokyo 192-0904 Japan; 2 Clinical Research Center for Medicine, International University of Health and Welfare, Akasaka 8-5-35, Minato-ku, Tokyo, 107-0052 Japan; 3 Saiseikai Toyama Hospital, Kusunoki 33-1, Toyama, 931-8533 Japan; 4 Department of Medicine and Clinical Science, Graduate School of Medical Sciences, Kyushu University, Maidashi 3-1-1, Higashi-ku, Fukuoka, 812-8582 Japan; 5 Cardiovascular Institute Hospital, Nishiazabu 3-2-19, Minato-Ku, Tokyo, 106-0031 Japan; 6 Department of Cardiovascular Medicine, Graduate School of Medicine, Nippon Medical School, Sendagi 1-1-5, Bunkyo-ku, Tokyo, 113-8602 Japan; 7 Department of Cardiovascular Medicine, Faculty of Medicine, Toho University, Omorinishi 5-21-16, Ota-ku, Tokyo, 143-8540 Japan; 8 Department of Health Care Administration and Management, Center for Cohort Study, Kyushu University Graduate School of Medical Sciences, Maidashi 3-1-1, Higashi-ku, Fukuoka, 812-8582 Japan; 9 Department of Cardiovascular Medicine, Graduate School of Medical Sciences, Kumamoto University, 2-39-1, Kurokami Chuo-ku, Kumamoto, 860-8555 Japan; 10 Division of Heart Rhythm, International University of Health and Welfare Hospital, Iguchi 537-3, Nasushiobara, Japan; 11 Division of Biostatistics and Clinical Epidemiology, University of Toyama Graduate School of Medicine, Sugitani 2630, Toyama, 930-0194 Japan; 12 Department of Cardiovascular Medicine, Tohoku University Graduate School of Medicine, Seiryomachi 1-1, Aoba-ku, Sendai, 980-8574 Japan; 13 International University of Health and Welfare, Kozunomori 4-3, Narita, 286-8686 Japan

**Keywords:** Non-valvular atrial fibrillation, Rivaroxaban, Renal dysfunction, Creatinine clearance

## Abstract

The EXPAND Study demonstrated the effectiveness and safety of rivaroxaban in patients with non-valvular atrial fibrillation (NVAF) in routine clinical practice in Japan. This sub-analysis was conducted to reveal the effectiveness and safety of rivaroxaban in Japanese NVAF patients according to baseline creatinine clearance (CrCl) levels and rivaroxaban doses in the EXPAND Study. We examined 6806 patients whose baseline CrCl data were available and classified them into 2 groups: normal renal function group with CrCl ≥ 50 mL/min (*n* = 5326, 78%) and renal dysfunction group with CrCl < 50 mL/min (*n* = 1480, 22%). In the normal renal function group, 1609 (30%) received 10 mg/day (under-dose), while in the renal dysfunction group, 108 (7%) received 15 mg/day (over-dose). In the normal renal function group, under-dose of rivaroxaban was associated with higher all-cause mortality, while in the renal dysfunction group, over-dose was associated with higher incidence of major bleeding. In contrast, the incidence of stroke or systemic embolism was not different between the 2 groups regardless of the dose of rivaroxaban. In the propensity score matched analysis to adjust the difference in characteristics according to doses of rivaroxaban, the incidences of clinical outcomes were comparable between the 2 dose groups in both renal function groups. These results indicate that the dose of rivaroxaban should be reduced depending on the renal function, considering the balance between risks of bleeding and ischemia.

## Introduction

Atrial fibrillation (AF) is a most commonly encountered arrhythmia in routine clinical practice and is widely known as a risk factor for not only stroke and systemic embolism (SE) but also dementia [1, 2]. In Japan, the morbidity of AF has been increasing along with rapid aging of the society [3]. Direct oral anticoagulants (DOACs) are widely used for the prevention of stroke/SE in patients with non-valvular atrial fibrillation (NVAF); however, they require dose adjustment according to renal function and are thus contraindicated in patients with severe renal dysfunction [4, 5]. In addition, aggravation of renal function with aging has been reported as in the case of glucose tolerance and lipid metabolism [6].

Renal dysfunction is an independent risk factor for ischemic stroke, and the risks of stroke and all-cause death are increased in patients with AF [7–13]. A previous meta-analysis reported that warfarin decreased the risk of ischemic stroke/SE and all-cause mortality in patients with non-end stage chronic kidney disease [14]. In addition, a relationship between renal dysfunction and clinical outcomes in Japanese patients with AF has been examined in the Fushimi AF Registry and the J-RHYTHM Registry [15, 16]; however, the evidence in patients treated with DOACs remains to be accumulated more.

The safety and efficacy of rivaroxaban, one of DOACs, were evaluated as compared with dose-adjusted warfarin in the previous clinical trials (ROCKET AF and J-ROCKET AF trials) [17, 18]. In the global ROCKET AF trial, rivaroxaban was administered at a dose of 20 mg/day and 15 mg/day to patients with normal renal function and those with renal dysfunction with a creatinine clearance (CrCl) ≤ 50 mg/min, respectively. The results showed no interaction between the study doses regardless of the state of renal function [17, 19]. In the J-ROCKET AF trial in Japan, which was conducted to examine the safety and efficacy of rivaroxaban at doses adjusted for Japanese patients, rivaroxaban was administered at a dose of 15 mg/day and 10 mg/day to patients with normal renal function and those with renal dysfunction, respectively [18]. There was no interaction in the safety or efficacy of rivaroxaban between the study doses regardless of the state of renal function as in the case of the ROCKET AF trial [18, 20].

The EXPAND Study was an investigator-initiated, multicenter registry to investigate the effectiveness and safety of rivaroxaban for the prevention of stroke/SE events in NVAF patients in routine clinical practice in Japan [21]. In the present sub-analysis, we aimed to identify the situation of rivaroxaban doses by renal function, and to reveal the effectiveness and safety of rivaroxaban in Japanese NVAF patients with renal dysfunction in the EXPAND Study.

## Methods

The EXPAND Study was conducted in accordance with the principles of the Declaration of Helsinki, the Ethical Guidelines for Clinical Studies from the Japanese Ministry of Health, Labour and Welfare, and all applicable laws and regulations in Japan [21]. The protocol was reviewed and approved by the Institutional Review Boards and/or Ethics Committee at all the participating institutes. All subjects provided written informed consent before enrollment in this study. The present study is registered with Clinical trials. gov., number NCT02147444, and with the University Hospital Medical Information Network Clinical Trials Registry, number UMIN000009376.

In the present sub-analysis, we included 6806 patients whose baseline CrCl data were available and divided them into 2 groups by baseline CrCl levels; normal renal function group with CrCl ≥ 50 mL/min and renal dysfunction group with CrCl < 50 mL/min. The 2 groups were further stratified by the dose of rivaroxaban (10 mg/day vs. 15 mg/day). The value of CrCl was estimated by Cockcroft-Gault formula as $\left( {140\; - \;\left[ {{\text{age}}} \right]} \right)\; \times \;\left( {{\text{body}}\;{\text{weight}}} \right)/72\; \times \;\left( {{\text{serum}}\;{\text{creatinine}}} \right)\;\left( { \times \;0.85\;{\text{for}}\;{\text{female}}s} \right)$ [22]. The patients treated with rivaroxaban 15 mg in the renal dysfunction group were defined as an over-dose group and those treated with rivaroxaban 10 mg in the normal renal function group was defined as an under-dose group. The 2 standard-dose groups were defined as the patients treated with rivaroxaban 10 mg and 15 mg in the renal dysfunction and the normal renal function groups, respectively.

Baseline patient characteristics were described and stratified by renal function and rivaroxaban dose. The cumulative incidences of stroke/SE, ischemic stroke, major bleeding, and non-major bleeding were evaluated and compared. Bleeding events were defined as International Society on Thrombosis and Haemostasis criteria [23]. The annual incidences of stroke/SE, ischemic stroke, all-cause death, major bleeding, and non-major bleeding were estimated based on the time to first event using the Kaplan–Meier estimate and calculated using the log-rank test by renal function and rivaroxaban dose.

Numerical data are expressed as mean ± standard deviation (SD). Testing for significant differences was performed by a *χ*^2^ test, Wilcoxon rank-sum test, or Student’s *t* test. We used propensity score matching (PSM) to adjust the clinical backgrounds between patients with normal renal function and those with renal dysfunction for the following clinical variables: age, sex, type of AF, body weight, congestive heart failure, hypertension, diabetes mellitus, ischemic stroke/SE, cardiovascular disease as a myocardial infraction/angina pectoris, CrCl level, history of bleeding/bleeding tendency, and concomitant use of anti-platelet agents. A two-sided significance level of 5% was employed, and all statistical analyses were conducted using SAS software (for Windows Release ver.9.2 or later versions; SAS Institute Inc.).

## Results

Of the 7141 patients enrolled in the EXPAND Study, 6806 patients with CrCl values at baseline were analyzed in the present study. A total of 5326 patients were included in the normal renal function group (CrCl ≥ 50 mL/min), and 1480 patients in the renal dysfunction group (CrCl < 50 mL/min) (Table 1). In the normal renal function group, 30.2% of the patients (1609/5326 patients) received the reduced dose of rivaroxaban 10 mg/day as an under-dose group, while in the renal dysfunction group, 7.3% of the patients (108/1480 patients) received the non-reduced dose of rivaroxaban 15 mg/day as an over-dose group (Table 1). The number of patients in the standard-dose groups was 92.7% of the renal dysfunction (1372/1480 patients) and 69.8% of the normal renal function (3717/5326 patients).

**Table 1:** Patient characteristics by creatinine clearance

	Total	Normal renal function group (CrCl ≥ 50 mL/min)	Renal dysfunction group (CrCl < 50 mL/min)	*P* value^†^
	(*n* = 6806)	(*n* = 5326)	(*n* = 1480)	
Sex (male), *n* (%)	4605 (67.7)	3838 (72.1)	767 (51.8)	< 0.001
Age (years), mean ± SD	71.6 ± 9.4	69.4 ± 8.9	79.8 ± 6.1	< 0.001
Age ≥ 75 years, *n* (%)	2819 (41.4)	1596 (30.0)	1223 (82.6)	< 0.001
Body weight (kg), mean ± SD	62.7 ± 12.5	65.5 ± 11.8	52.9 ± 9.5	< 0.001
CHADS_2_ score, mean ± SD	2.1 ± 1.3	1.9 ± 1.3	2.8 ± 1.3	< 0.001
< 2 points, *n* (%)	2498 (36.7)	2279 (42.8)	219 (14.8)	< 0.001
2 points, *n* (%)	1990 (29.2)	1510 (28.4)	480 (32.4)	
≥ 3 points, *n* (%)	2318 (34.1)	1537 (28.9)	781 (52.8)	
CHA_2_DS_2_-VASc score, mean ± SD	3.4 ± 1.7	3.1 ± 1.6	4.5 ± 1.5	< 0.001
HAS-BLED score, mean ± SD	1.4 ± 0.9	1.4 ± 0.9	1.6 ± 0.8	< 0.001
Rivaroxaban dosage, *n* (%)				
10 mg/day	2981 (43.8)	1609 (30.2)	1372 (92.7)	–
15 mg/day	3825 (56.2)	3717 (69.8)	108 (7.3)	–
Comorbidity and medical history, *n* (%)				
Congestive heart failure	1806 (26.5)	1209 (22.7)	597(40.3)	< 0.001
Hypertension	4843 (71.2)	3743 (70.3)	1100 (74.3)	0.002
Diabetes mellitus	1679 (24.7)	1323 (24.8)	356 (24.1)	0.535
Angina pectoris	803 (11.8)	582 (10.9)	221 (14.9)	< 0.001
Dyslipidemia	2864 (42.1)	2270 (42.6)	594 (40.1)	0.087
Stroke (ischemic/hemorrhagic)	1459 (21.4)	1068 (20.1)	391 (26.4)	< 0.001
Ischemic stroke	1373 (20.2)	1001 (18.8)	372 (25.1)	< 0.001
Hemorrhagic stroke	130 (1.9)	98 (1.8)	32 (2.2)	0.423
Transient ischemic attack	206 (3.0)	152 (2.9)	54 (3.6)	0.114
Systemic embolism	58 (0.9)	43 (0.8)	15 (1.0)	0.445
Myocardial infarction	285 (4.2)	199 (3.7)	86 (5.8)	< 0.001
Malignant tumor	625 (9.2)	446 (8.4)	179 (12.1)	< 0.001
Bleeding and/or bleeding tendency	276 (4.1)	218 (4.1)	58 (3.9)	0.764
Non-PAF (persistent/permanent), *n* (%)	3783 (55.6)	2922 (54.9)	861 (58.2)	0.023
Use of concomitant anti-platelet, *n* (%)	640 (9.4)	727 (13.7)	263 (17.8)	< 0.001

*CrCl* creatinine clearance, *PAF* paroxysmal atrial fibrillation, *SD* standard deviation

^†^CrCl ≥ 50 mL/min vs. CrCl < 50 mL/min

### Baseline patient characteristics by renal function

Patient characteristics in the normal renal function and renal dysfunction groups are shown in Table 1. There were significant differences in most factors at baseline between the 2 groups.

Patient characteristics by CrCl and rivaroxaban dose at baseline are shown in Table 2. In unmatched cohort, in the renal dysfunction group, there was no difference in clinical characteristics except for mean age and proportion of age ≥ 75 years between the standard-dose and over-dose group. In contrast, in the normal renal function group, there were significant differences in the majority of factors between the standard-dose and the under-dose group, except for diabetes mellitus, dyslipidemia, stroke including ischemic and hemorrhagic, SE, and non-paroxysmal AF.

**Table 2:** Patient characteristics by creatinine clearance and rivaroxaban doses in the unmatched and propensity score matched cohorts

	Unmatched cohort						Propensity Score Matched cohort					
	Normal renal function group (CrCl ≥ 50 mL/min)			Renal dysfunction group (CrCl < 50 mL/min)			Normal renal function group (CrCl ≥ 50 mL/min)			Renal dysfunction group (CrCl < 50 mL/min)		
	10 mg/day	15 mg/day	*P* value^†^	10 mg/day	15 mg/day	*P* value^†^	10 mg/day	15 mg/day	*P* value^†^	10 mg/day	15 mg/day	*P* value^†^
	(*n* = 1609)	(*n* = 3717)		(*n* = 1372)	(*n* = 108)		(*n* = 1314)	(*n* = 1314)		(*n* = 108)	(*n* = 108)	
Sex (male), *n* (%)	1025 (63.7)	2813 (75.7)	< 0.001	711 (51.8)	56 (51.9)	0.995	886 (67.4)	874 (66.5)	0.619	60 (55.6)	56 (51.9)	0.585
Age (years), mean ± SD	73.8 ± 7.8	67.4 ± 8.6	< 0.001	80.0 ± 6.1	77.0 ± 6.0	< 0.001	72.6 ± 7.3	72.6 ± 6.6	0.898	77.9 ± 6.0	77.0 ± 6.0	0.258
Age ≥ 75 years, *n* (%)	829 (51.5)	767 (20.6)	< 0.001	1147 (83.6)	76 (70.4)	< 0.001	570 (43.4)	567 (43.2)	0.906	75 (69.4)	76 (70.4)	0.882
Body weight (kg), mean ± SD	62.8 ± 10.9	66.6 ± 12.0	< 0.001	52.9 ± 9.6	52.4 ± 7.9	0.223	63.2 ± 10.9	63.4 ± 11.0	0.631	53.0 ± 8.0	52.4 ± 7.9	0.620
CHADS_2_ score, mean ± SD	2.2 ± 1.3	1.8 ± 1.2	< 0.001	2.8 ± 1.3	2.6 ± 1.3	0.147	2.1 ± 1.3	2.1 ± 1.3	0.867	2.4 ± 1.3	2.6 ± 1.3	0.311
< 2 points, *n* (%)	521 (32.4)	1758 (47.3)	< 0.001	198 (14.4)	21 (19.4)	0.055	493 (37.5)	455 (34.6)	0.339	27 (25.0)	21 (19.4)	0.602
2 points, *n* (%)	496 (30.8)	1014 (27.3)	–	441 (32.1)	39 (36.1)	–	385 (29.3)	421 (32.0)	-	34 (31.5)	39 (36.1)	–
≥ 3 points, *n* (%)	592 (36.8)	945 (25.4)	–	733 (53.4)	48 (44.4)	–	436 (33.2)	438 (33.3)	-	47 (43.5)	48 (44.4)	–
CHA_2_DS_2_-VASc score, mean ± SD	3.7 ± 1.6	2.8 ± 1.6	< 0.001	4.5 ± 1.5	4.3 ± 1.5	0.169	3.5 ± 1.5	3.5 ± 1.5	0.604	4.0 ± 1.3	4.3 ± 1.5	0.172
HAS-BLED score, mean ± SD	1.5 ± 0.9	1.3 ± 0.9	< 0.001	1.6 ± 0.8	1.5 ± 0.8	0.298	1.5 ± 0.9	1.5 ± 0.9	0.400	1.7 ± 0.8	1.5 ± 0.8	0.191
Comorbidity and medical history, *n* (%)												
Congestive heart failure	449 (27.9)	760 (20.4)	< 0.001	560 (40.8)	37 (34.3)	0.181	335 (25.5)	345 (26.3)	0.656	36 (33.3)	37 (34.3)	0.886
Hypertension	1213 (75.4)	2530 (68.1)	< 0.001	1017 (74.1)	83 (76.9)	0.532	965 (73.4)	974 (74.1)	0.690	76 (70.4)	83 (76.9)	0.280
Diabetes mellitus	406 (25.2)	917 (24.7)	0.663	332 (24.2)	24 (22.2)	0.644	323 (24.6)	334 (25.4)	0.620	18 (16.7)	24 (22.2)	0.302
Angina pectoris	239 (14.9)	343 (9.2)	< 0.001	209 (15.2)	12 (11.1)	0.247	170 (12.9)	159 (12.1)	0.517	14 (13.0)	12 (11.1)	0.676
Dyslipidemia	704 (43.8)	1566 (42.1)	0.271	552 (40.2)	42 (38.9)	0.784	565 (43.0)	563 (42.8)	0.937	42 (38.9)	42 (38.9)	1.000
Stroke (ischemic/hemorrhagic)	306 (19.0)	762 (20.5)	0.215	363 (26.5)	28 (25.9)	0.904	252 (19.2)	245 (18.6)	0.727	28 (25.9)	28 (25.9)	1.000
Ischemic stroke	285 (17.7)	716 (19.3)	0.184	347 (25.3)	25 (23.1)	0.621	231 (17.6)	221 (16.8)	0.605	26 (24.1)	25 (23.1)	0.873
Hemorrhagic stroke	28 (1.7)	70 (1.9)	0.721	28 (2.0)	4 (3.7)	0.253	28 (2.1)	30 (2.3)	0.791	2 (1.9)	4 (3.7)	0.408
Transient ischemic attack	58 (3.6)	94 (2.5)	0.030	48 (3.5)	6 (5.6)	0.272	47 (3.6)	43 (3.3)	0.668	5 (4.6)	6 (5.6)	0.757
Systemic embolism	12 (0.7)	31 (0.8)	0.741	14 (1.0)	1 (0.9)	0.925	11 (0.8)	7 (0.5)	0.344	0 (0.0)	1 (0.9)	1.000
Myocardial infarction	84 (5.2)	115 (3.1)	< 0.001	81 (5.9)	5 (4.6)	0.586	58 (4.4)	57 (4.3)	0.924	1 (0.9)	5 (4.6)	0.098
Malignant tumor	157 (9.8)	289 (7.8)	0.017	162 (11.8)	17 (15.7)	0.227	128 (9.7)	124 (9.4)	0.791	11 (10.2)	17 (15.7)	0.224
Bleeding and/or bleeding tendency	82 (5.1)	136 (3.7)	0.015	55 (4.0)	3 (2.8)	0.526	57 (4.3)	64 (4.9)	0.515	7 (6.5)	3 (2.8)	0.195
Non-PAF (persistent/permanent), *n* (%)	889 (55.3)	2033 (54.7)	0.708	803 (58.5)	58 (53.7)	0.328	739 (56.2)	732 (55.7)	0.783	55 (50.9)	58 (53.7)	0.683
Use of concomitant antiplatelet, *n* (%)	284 (17.7)	443 (11.9)	< 0.001	246 (17.9)	17 (15.7)	0.567	210 (16.0)	213 (16.2)	0.874	20 (18.5)	17 (15.7)	0.588

*CrCl* creatinine clearance, *PAF* paroxysmal atrial fibrillation, *SD* standard deviation

^†^10 mg/day vs. 15 mg/day

In the PSM analysis, patient characteristics were well balanced between rivaroxaban 10 mg/day and 15 mg/day groups in both renal function groups, and those results are shown as propensity score matched cohort in Table 2.

### Effectiveness and safety endpoints

The effectiveness and safety outcomes by CrCl are shown in Table 3. The incidence of stroke/SE was significantly higher in the renal dysfunction group than in the normal renal function group (1.47%/year vs. 0.85%/year, *P* < 0.001), and this was also the case for the incidence of ischemic stroke (1.13%/year vs. 0.61%/year, *P* = 0.001) and all-cause death (3.79%/year vs. 1.03%/year, P < 0.001). Regarding the safety, the incidence of major bleeding was significantly higher in the renal dysfunction group than in the normal renal function group (1.92%/year vs. 1.05%/year, *P* < 0.001), while there was no significant difference in the incidence of non-major bleeding (5.15%/year vs. 4.80%/year, *P* = 0.474) between the 2 groups.

**Table 3:** Effectiveness and safety endpoints by creatinine clearance

	Total	Normal renal function group (CrCl ≥ 50 mL/min)	Renal dysfunction group (CrCl < 50 mL/min)	*P* value^†^
	(*n* = 6806)	(*n* = 5326)	(*n* = 1480)	
Effectiveness endpoint				
Stroke/Systemic embolism	164 (0.98)	112 (0.85)	52 (1.47)	< 0.001
Ischemic stroke	121 (0.72)	81 (0.61)	40 (1.13)	0.001
All-cause death	270 (1.61)	136 (1.03)	134 (3.79)	< 0.001
Safety endpoint				
Major bleeding	206 (1.23)	138 (1.05)	68 (1.92)	< 0.001
Non-major bleeding	815 (4.87)	633 (4.80)	182 (5.15)	0.474

Figures are number of event (%/year)

*CrCl* creatinine clearance

^†^CrCl ≥ 50 mL/min vs. CrCl < 50 mL/min

**Fig. 1:**
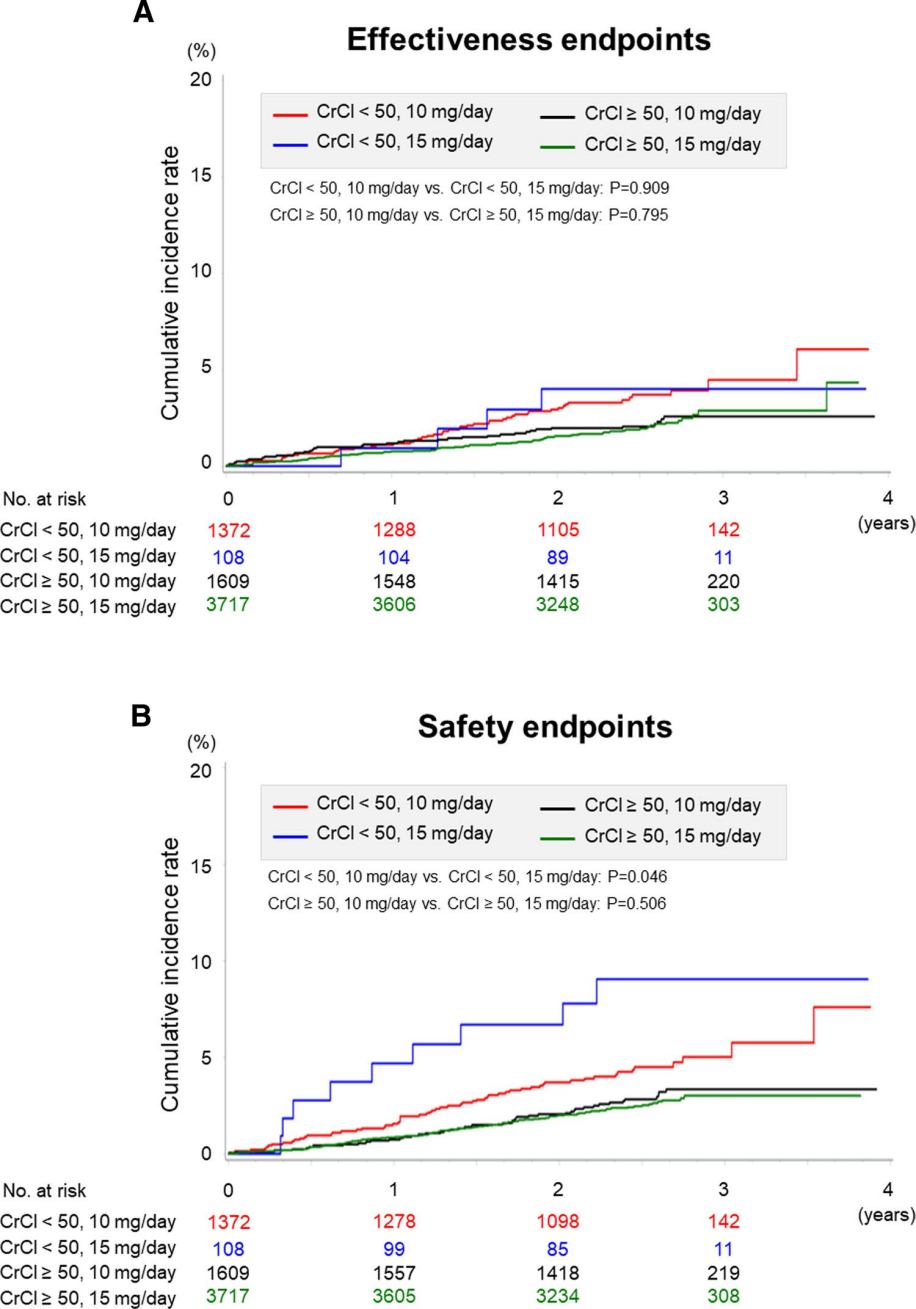
Kaplan–Meier estimates for the primary effectiveness endpoints (**a**) and safety endpoints (**b**) by creatinine clearance and rivaroxaban doses in the unmatched cohort. CrCl; creatinine clearance

The effectiveness and safety outcomes according to CrCl and rivaroxaban doses in the unmatched cohort are shown in Fig. 1 and Table 4. In the renal dysfunction group, there was significant difference in the incidence of major bleeding event between standard-dose and over-dose groups (1.80%/year vs. 3.53%/year, *P* = 0.046). In the normal renal function group, there was no significant difference in the incidence of major bleeding event between under-dose and standard-dose groups (1.13%/year vs. 1.01%/year, P = 0.506), whereas significant difference in the incidence of all-cause death was noted between the 2 groups (1.67%/year vs. 0.75%/year, *P* < 0.001).

**Table 4:** Effectiveness and safety endpoints by creatinine clearance and rivaroxaban doses in the unmatched and propensity score matched cohorts

	Unmatched cohorts						Propensity score matched cohorts						
	Normal renal function group (CrCl ≥ 50 mL/min)			Renal dysfunction group (CrCl < 50 mL/min)			Normal renal function group (CrCl ≥ 50 mL/min)			Renal dysfunction group (CrCl < 50 mL/min)			
	10 mg/day	15 mg/day	*P* value^†^	10 mg/day	15 mg/day	*P* value^†^	10 mg/day	15 mg/day	*P* value^†^		10 mg/day	15 mg/day	*P* value^†^
	(*n* = 1609)	(*n* = 3717)		(*n* = 1372)	(*n* = 108)		(*n* = 1314)	(*n* = 1314)			(*n* = 108)	(*n* = 108)	
Effectiveness endpoint													
Stroke/Systemic embolism	36 (0.88)	76 (0.83)	0.795	48 (1.46)	4 (1.57)	0.909	32 (0.96)	27 (0.84)	0.595		2 (0.74)	4 (1.57)	0.387
Ischemic stroke	26 (0.64)	55 (0.60)	0.816	36 (1.10)	4 (1.57)	0.512	23 (0.69)	21 (0.65)	0.834		2 (0.74)	4 (1.57)	0.387
All-cause death	68 (1.67)	68 (0.75)	< 0.001	121 (3.69)	13 (5.09)	0.243	46 (1.38)	31 (0.96)	0.146		7 (2.60)	13 (5.09)	0.123
Safety endpoint													
Major bleeding	46 (1.13)	92 (1.01)	0.506	59 (1.80)	9 (3.53)	0.046	31 (0.93)	45 (1.40)	0.082		3 (1.11)	9 (3.53)	0.067
Non-major bleeding	177 (4.34)	456 (5.00)	0.988	168 (5.12)	14 (5.48)	0.769	143 (4.28)	181 (5.62)	0.010		8 (2.97)	14 (5.48)	0.148

Figures are number of event (%/year)

*CrCl* creatinine clearance

^†^10 mg/day vs. 15 mg/day

**Fig. 2:**
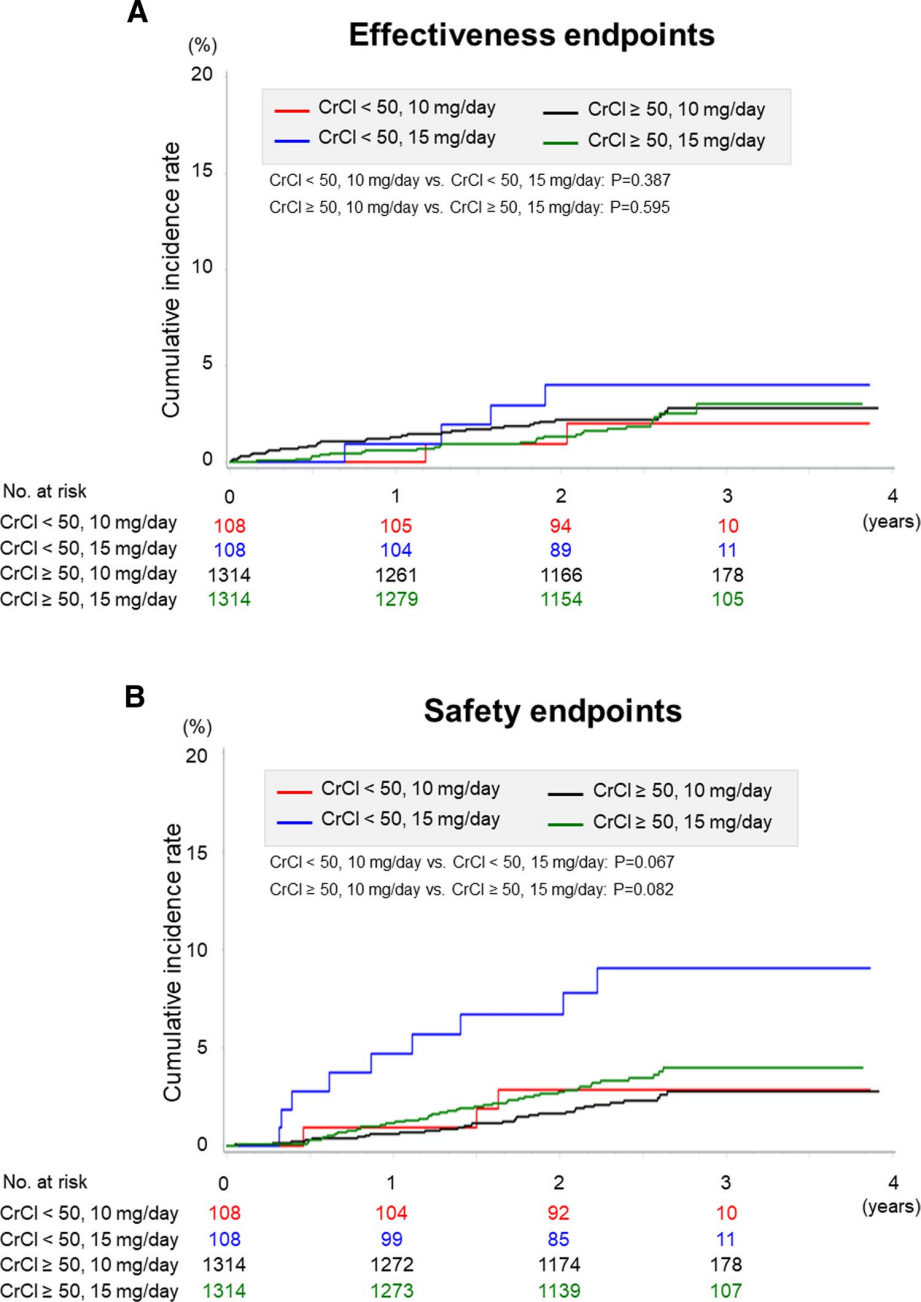
Kaplan–Meier estimates for the primary effectiveness endpoints (**a**) and safety endpoints (**b**) by creatinine clearance and rivaroxaban doses in the propensity score matched cohort. CrCl; creatinine clearance

The effectiveness and safety endpoints according to CrCl and rivaroxaban doses in the propensity score matched cohort are shown in Fig. 2 and Table 4. In the renal dysfunction group, although not significantly different, the incidence rates of all endpoints tended to be higher in the over-dose than in the standard-dose groups. In contrast, in the normal renal function group, there was a significant difference in non-major bleeding event between the under-dose and the standard-dose groups (4.28%/year vs. 5.62%/year, *P* = 0.010).

## Discussion

The present study was conducted to reveal the actual conditions of use and the effectiveness and safety of rivaroxaban in Japanese NVAF patients with a special reference to renal function in the EXPAND Study. In the normal renal function group, 30% received under-dose, and in the renal dysfunction group, 7% received over-dose of rivaroxaban. In the renal dysfunction group, the incidence of stroke/SE was higher than in the normal renal function group and the incidences of ischemic stroke and all-cause death were also higher in the former than in the latter, which was also the case for major bleeding.

The incidences of stroke and major bleeding have been reported to elevate due to renal dysfunction even without anticoagulation [6–13]. Although warfarin has been reported to decrease the risk in patients at risk of thromboembolism [11, 24], this point remains to be adequately evaluated for DOACs. For the clinical issue of renal dysfunction, poor control of time in therapeutic range is known to be a poor prognostic factor for the use of warfarin [25]. DOACs could solve this issue if adherence is maintained. However, not only the risk of stroke/SE but also the risk of bleeding is anticipated to increase in patients with renal dysfunction. In a study using a database in the UK with PSM analysis [26], anticoagulant therapy was found to be associated with increased rate of ischemic stroke and hemorrhage but with lower all-cause mortality in elderly patients aged ≥ 65 years with reduced estimated glomerular filtration rate [26]. In this UK study, 71.7% of the patients were on warfarin, an approximately one-quarter of all the patients used DOACs, of whom 12.7% were on rivaroxaban [26].

For the risk assessment in NVAF patients, the CHADS_2_ and CHA_2_DS_2_-VASc scores are widely used, and important risk of anticoagulation is bleeding. The present results also support the importance of adjustment of dose of rivaroxaban based on renal dysfunction as evaluated by CrCl values. The ORBIT bleeding score proposes an evaluation method adding renal dysfunction (hazard ratio 1.44) [27]. In the current era with DOACs, it is important to add renal function for evaluation of bleeding risk. For those with CrCl < 50 mL/min, as demonstrated in the present study, the bleeding risk with rivaroxaban should be adequately assessed, and the clinical course should also be carefully monitored.

It is recommended to decrease the dose of rivaroxaban for patients with renal dysfunction, although it has been reported that in real-world clinical practice, doses are often chosen that are not as described in the package insert, considering the individual patient's background [15, 16, 28]. In the present study, there were a small number of patients receiving over-dose of rivaroxaban, and its influence was compared with those receiving standard-dose in the unmatched and the propensity score matched cohorts. The results showed that the incidence of stroke/SE in the renal dysfunction group did not differ between standard-dose and over-dose in the unmatched cohorts, whereas the incidence of major bleeding increased in this group with over-dose in the unmatched cohort. In contrast, interestingly, the incidence of stroke/SE tended to be higher and that of major bleeding tended to be lower in the under-dose group as compared with the standard-dose group in the propensity score matched cohort. The XAPASS showed that the under-dosing was associated with a decreased incidence rate of any bleeding, but not that of major bleeding [29]. In the normal renal function group, there were significant differences between patients with standard-dose and those with under-dose in characteristics at baseline, CHADS_2_, CHA_2_DS_2_-VASc, and HAS-BLED scores. The patients with low CHADS_2_ score as a less than 2 points were included approximately 32% and 47% in the under-dose and standard-dose groups, respectively. In our previous report of this EXPAND study, we showed that patients with a higher CHADS_2_ score had a higher incidence rate of stroke/SE and major bleeding events [21]. However, in the results of the present sub-analysis of unmatched cohort, there was no change in the incidence rates except for all-cause death between the under-dose and standard-dose groups despite the fact that mean CHADS_2_ score was higher in the under-dose than in the standard-dose groups. Physicians are reducing dose of anticoagulants for elderly AF patients based on the risk, such as frailty, polypharmacy, and dementia or cognitive impairment [30–32], which impossible to express by the CHADS_2_ score [33]. Under dosing of anticoagulant for elderly patients may be superior to standard dosing [34], and the results of this study seem to support those findings.

As for the reason for the higher mortality in the under-dose group may be due to the fact that the under-dose group included more elderly patients as compared with the standard-dose group. On the other hand, the GARFIELD-AF study showed that under-dosing of DOACs was associated with an increased risk of death, and under-dosing was associated with higher risk of death compared with standard dosing (hazard ratio 1.25, 95% CI 1.04–1.50) [35]. In this registry, the patients’ background, such as age, and CHA_2_DS_2_-VASc score and the incidence of stroke and bleeding, was similar to those of the EXPAND Study. However, this registry included a variety of races, and it is necessary to consider racial differences and Japan-specific dose of DOACs.

With regard to the incidence of major bleeding, the propensity score matched cohort in the normal renal function group showed a numerically higher incidence in the rivaroxaban standard-dose than in the under-dose groups, with no difference in ischemic events. These results indicate that the dose should be decreased in accordance with the criteria for dose reduction for patients with renal dysfunction (10 mg for patients with CrCl < 50 mL/min) and suggest that some NVAF patients with normal renal function may do better with under-dose of rivaroxaban considering the balance between the risks of bleeding and ischemic events.

### Study limitations

We have previously mentioned several limitations in the EXPAND Study [21, 36, 37]. Furthermore, in the present sub-analysis, the following limitations should be considered. First, although patients were classified by CrCl values and the dose of rivaroxaban at baseline, CrCl values and the dose of rivaroxaban at the onset of events were not available. Second, since the EXPAND Study was an observational study, the dose of rivaroxaban was chosen at the discretion of physicians, and no assessment of adherence was performed. Third, the analysis of this report used propensity score matching, and the number of patients after the matching was decreased by 85% in the renal dysfunction group and by 51% in the normal renal function groups. This could have affected the results of the present sub-analysis. Finally, a relatively small number of endpoints might have failed to detect a clinically important difference in safety.

## Conclusion

In the present sub-analysis of the EXPAND Study, we were able to demonstrate the incidences of effectiveness and safety events in NVAF patients according to renal function and rivaroxaban doses. The present results support the current recommendation that the dose of rivaroxaban should be reduced to 10 mg/day for patients with renal dysfunction with CrCl < 50 mL/min. On the other hand, rivaroxaban dose should be reduced depending on renal function, considering the balance between risks of bleeding and ischemia. However, since there was no enough evidence for using under-dose in patients with normal renal function, further clinical data are needed for better use of rivaroxaban in those patients.

## Abbreviations

AF: Atrial fibrillation
CrCl: Creatinine clearance
DOACs: Direct oral anticoagulants
NVAF: Non-valvular atrial fibrillation
PSM: Propensity score matching
SD: Standard deviation
SE: Systemic embolism
